# Immunomodulatory Effects of Drugs for Effective Cancer Immunotherapy

**DOI:** 10.1155/2018/8653489

**Published:** 2018-10-25

**Authors:** Maiko Matsushita, Mai Kawaguchi

**Affiliations:** Division of Clinical Physiology and Therapeutics, Keio University, Faculty of Pharmacy, 1-5-30, Shiabakoen, Minato-Ku, Tokyo, Japan

## Abstract

Recent advances in cancer immunotherapy, including immune checkpoint inhibitors or adoptive T cell therapies, have contributed to better outcomes in cancer patients. However, there are still many cancers with no cure. Therefore, combinations of several treatment strategies are being explored, and enhancing anticancer immunity will play an important role to combat the disease. There have been several reports on the immune-modulatory effects of commonly used drugs, namely, statin, metformin, and angiotensin receptor blockers (ARBs), which suggest that these drugs could enhance immunity against cancer cells. Other anticancer drugs, such as anthracyclines, thalidomides, lenalidomides, and hypomethylating drugs, could also strengthen the immune system to attack cancer cells at a relatively low dose. Hence, these drugs might contribute to better outcomes in cancer patients.

## 1. Introduction

Immune system dysfunction is related to many diseases, including inflammatory diseases, autoimmune diseases, infectious diseases, atherosclerosis, and cancer [1–5]. Several drugs that directly target the immune system have been developed for the treatment of these disorders. For example, immune checkpoint inhibitors (ICIs) have greatly improved the outcome of various cancers by altering patients' immune-suppressive status and enhancing antitumor immunity [6–8]. However, even ICIs are ineffective in the treatment and cure of certain cancers. Therefore, new strategies to enhance the efficacy of these treatments are needed. Moreover, ICIs and many of the other targeted anticancer therapies consist of monoclonal antibodies, which make them highly expensive.

On the other hand, immunomodulatory effects have been described for several small molecular drugs, which have been used for a long time to treat common diseases, including hyperlipidemia, diabetes, and hypertension [9–11]. These drugs are cost-effective and could be used safely, as their adverse reactions are well known. If immunomodulatory effects of these previously approved drugs are clearly established, it might be possible to enhance the effects of conventional cancer therapy by combinatorial use of these drugs [12, 13]. In this review, we discuss drugs that have been reported to elicit immunomodulatory effects in addition to their original pharmacological effects.

We searched previously published literature for papers on immunomodulatory function of drugs using PubMed (https://www.ncbi.nlm.nih.gov/pubmed/). By using “immunomodulating” and “drugs” as search words, we obtained 3433 papers (August 2018). We excluded papers with monoclonal antibodies and previously approved immunosuppressive drugs, such as corticosteroids. We classified the remaining drugs, obtained in the search, based on their effects on cytokines (Table 1), immune cells (T cells, B cells, and antigen presenting cells; Table 2), and immune-related signaling pathways (nuclear factor-kappa B: NF-kB, Signal Transducers and Activator of Transcription: STAT, Peroxisome Proliferator-Activated Receptor *γ*: PPAR1*γ*, Extracellular Signal-Regulated Kinase: ERK, mechanistic target of rapamycin: mTOR, and AMP-activated protein kinase: AMPK; Table 3).

To ascertain drugs that could be used for cancer treatment, we selected the drugs that have been reported to have anticancer effects by enhancing immunological responses to cancer cells. Three drugs approved for treatment of common diseases (statin, metformin, and angiotensin receptor blocker) and three categories of anticancer drugs that show immunomodulatory effects (thalidomide and its derivatives, anthracyclines or other chemotherapeutic drugs, and demethylating agents) are discussed further for their anticancer property via immunological mechanisms.

## 2. Immunomodulatory Effects of Drugs Used for Treating Common Diseases

### 2.1. Statins

Statins have been used by millions of people for lowering blood lipids to prevent coronary heart disease [14]. They inhibit hydroxymethylglutaryl (HMG) CoA reductase in the cholesterol biosynthesis pathway. In addition to their cholesterol-lowering effects, many studies have shown that statins also exhibit immune-modulatory properties through mevalonate-independent and -dependent pathways [15]. They inhibit the activity of Ras and Rho family GTPases, which regulate various cellular functions, such as cell death, metastasis, and immune reactions [16–18]. Statins affect the immune cells by decreasing the production of inflammatory cytokines and activating CD8^+^ T cells [19]. Sarrabayrouse et al. have also reported that statin treatment induces the expression of CD80 and CD86 in melanoma cells by upregulating their gene expression through transcriptional factors controlled by Rho proteins [20]. In fact, clinical studies have confirmed the role of statins in preventing several types of cancer [21–23]. These data suggest that statins might be useful for enhancing the immunity against cancer cells.

### 2.2. Metformin

Metformin is one of the widely used antidiabetic drugs synthesized from the plant* Galega officinalis*, which lowers blood glucose level by suppressing hepatic glucose production in patients with type 2 diabetes [24]. It inhibits NADH: ubiquinone oxidoreductase located in the mitochondrial membrane, leading to the activation of 5′-AMP-activated protein kinase (AMPK) and suppression of gluconeogenesis. AMPK-independent pathway also contributes to its pharmacological actions [25]. In addition to its antihyperglycemic effects, there are many reports of its role in immune response. It has been shown that metformin enhances the number and function of tumor-infiltrating lymphocytes (TILs) [26]. Pereira et al. have reported that metformin exerts strong immunomodulatory effects and contributes to reduced lung metastasis of melanoma cells [27]. Cha et al. have shown that metformin reduces the stability and membrane localization of programmed death-ligand 1 (PD-L1) and contributes to the enhancement of cytotoxic T lymphocyte (CTL) activity against cancer cells [28]. Metformin also exerts anti-inflammatory effects [29, 30]. It was recently reported that these effects might be related to the alteration of gut microbiota [31]. These results suggest that metformin can be used for the treatment of cancer.

### 2.3. Angiotensin Receptor Blockers (ARBs)

Renin-angiotensin system mainly controls blood pressure; however, cancer cells and their microenvironment also express renin and angiotensin, which play a pathophysiological role in cancer development [32]. Several clinical studies have demonstrated that taking ARBs could decrease the risk of developing cancers [33, 34].* In vitro* and* in vivo* studies have further revealed that these anticancer effects of ARB include direct suppression of cancer growth and increase of antitumor immunity [35–38]. Nakamura et al. showed that administration of ARB to colon cancer-bearing mice induced the expansion of cancer-specific CTLs and reduced the levels of immune-suppressive cytokines, interleukin-6 (IL-6), IL-10, vascular endothelial cell factor, and arginase in the tumor microenvironment [39]. They demonstrated that combination therapy with ARB and anti PD-L1 antibody significantly reduced tumor size, suggesting that this therapy is also useful in clinical setting.

## 3. Immunomodulating Effects of Anticancer Drugs

### 3.1. Thalidomide, Lenalidomide, and Pomalidomide

Thalidomide, and its derivatives, lenalidomide, and pomalidomide are used as key drugs in the treatment of multiple myeloma, which is a plasma cell neoplasm [40–42]. Thalidomide first made headlines for its direct tumoricidal effects on myeloma cells by causing cell cycle arrest and also its antiangiogenic properties. Later, it was classified as an immunomodulatory drug (IMiD) owing to its immunological effects [43]. IMiDs stimulate T cells and natural killer T (NKT) cells to secrete IL-2 and interferon-*γ*, leading to NK cell activation and inhibition of regulatory T cells. As a result, myeloma-specific immunity is amplified [44–46]. The FDA has recently approved lenalidomide for maintenance therapy of posttransplant myeloma patients, considering that lenalidomide can suppress the residual myeloma cells.

### 3.2. Anthracyclines and Other Chemotherapeutic Drugs

Some of conventional anticancer drugs, such as anthracyclines or alkylating agents, have been reported to cause induction of immunomodulatory effects on various cancer cells by enhancing the cell surface expression of calreticulin (CRT) followed by the release of high mobility group box 1 (HMGB1), ATP, annexin A1, and type I interferon from cancer cells [47–50]. The dendritic cells (DCs) then recognize CRT, HMGB1, and ATP through CD91, Toll-like receptor 4, and P2X purinoreceptor 7, respectively, and take up the cancer cells. This series of events is called immunogenic cell death (ICD) [51–53]. Mouse immunization experiments using cancer cells pretreated with chemotherapeutic drugs, such as doxorubicin or mitoxantrone, have shown effective cancer regression through CRT expression and HMGB1 secretion [54–56]. These observations were further confirmed by clinical data, which have indicated that CRT expression is important for the improvement of disease outcome in cancer patients [57]. These results suggest that some of the chemotherapeutic drugs not only kill cancer cells directly but also strengthen patients' immune reactions against cancer cells, thereby contributing to the eradication of cancer cells. Moreover, other types of anticancer drugs, such as epidermal growth factor receptor inhibitors, also elicit immune reactions against cancer cells through ICD [58]. ICD inducers, especially cyclophosphamide, are now expected to enhance the antitumor effects of other immunotherapies, such as cancer vaccines or immune checkpoint inhibitors [59–62].

### 3.3. Azacitidine and Decitabine

Hypermethylation of tumor suppressor genes in the promoter region and subsequent silencing of these genes is observed in many cancer cells. Hence, hypomethylating agents (HMAs) are expected to be useful in cancer treatment by causing epigenetic modulation of these cancer-related genes [63]. HMAs, such as azacitidine or decitabine, have been approved for the treatment of myelodysplastic syndromes (MDS) and acute myeloid leukemia (AML) [64]. These HMAs are analogues of pyrimidine nucleosides, which are incorporated into RNA or DNA, and they impair DNA methylation by inhibiting DNA methyltransferase [65, 66]. At the same time, HMAs are known to enhance expression of cancer-specific antigens and MHC molecules, making cancer cells sensitive to killing by CTLs* in vitro* and* in vivo* [67–69]. Stone et al. have recently reported that HMAs could also alter immunosuppressive tumor microenvironment by activating type 1 interferon signaling, in combination with another class of epigenetic drugs, histone deacetylase inhibitors [70].

## 4. Conclusion

There have been reports on immunomodulating properties of several commonly used drugs, such as statins, metformin, and ARB, which can be used to treat or prevent cancer via increased antitumor immunity. These drugs can be used safely because of their known adverse reactions and might contribute to cancer treatment. Among anticancer drugs, anthracyclines, IMiDs, and epigenetic drugs not only kill the cancer cells directly, but also enhance the immune system to attack the cancer cells. It will be beneficial to combine these drugs with conventional cancer therapies to achieve better outcomes in cancer patients.

## Figures and Tables

**Table 1:** Effects on cytokine productions by commonly used drugs.

	IL-1	IL-6	IL-8	TNF-*α*	IFN-*γ*	IL-2	IL-4	IL-12	IL-18	TNF-*β*	IL-3	IL-5	IL-10	IL-13
H2 receptor antagonist														
NSAIDs														
Prostaglandin														
Norfloxacin		↓		↓									↑	
Linezolid	↓	↓	↓	↓	↓								↑	
Macrolide	↓		↓or↑	↑	↓	↓	↓or↑		↓or↑			↓or↑		↓or↑
Antifungal drugs		↑			↑	↑				↓			↓	
*β*2 adrenalin agonist														
D2 receptor agonist														
ACE inhibitor				↓										
ARB				↓	↓									
Ca channel blocker	↓or↑	↓or↑		↓	↓	↑								
Digoxin														
DPP4 inhibitor														
GLP-1 receptor agonist					↓		↓							
Biguanide														
Statin				↓										

**Table 2:** Effects on immune cells by commonly used drugs.

	T celll	Th1	Treg	Th17	MDSC	CTL	B cell	NK	CD4	CD8+TIL
H2 receptor antagonist	↑									
NSAIDs									↑	
Prostaglandin										
Norfloxacin										
Linezolid										
Macrolide									↑	
Antifungal drugs										
*β*2 adrenalin agonist										
D2 receptor agonist										
ACE inhibitor	↓									
ARB						↓				
Ca channel blocker										
Digoxin				↓						
DPP4 inhibitor			↑						↓	
Biguanide										↑
Statin	↓	↓	↑	↓				↓		

**Table 3:** Effects on gene expressions in immune-related signaling pathways by commonly used drugs.

	NF-*κ*B	STAT	PPAR1*γ*	ERK	mTOR	AMPK
H2 receptor antagonist						
NSAIDs						
Prostaglandin						
Norfloxacin						
Linezolid		↓				
Macrolide	↓			↓		
Antifungal drugs						
*β*2 adrenalin agonist						
D2 receptor agonist						
ACE inhibitor						
ARB						
Ca channel blocker	↓or↑					
Digoxin						
DPP4 inhibitor	↑					
Biguanide					↓	↑
Statin		↓	↑	↑		
Cannabidiol						
